# Generation of Pseudo-CT using High-Degree Polynomial Regression on Dual-Contrast Pelvic MRI Data

**DOI:** 10.1038/s41598-020-64842-3

**Published:** 2020-05-15

**Authors:** Samuel C. Leu, Zhibin Huang, Ziwei Lin

**Affiliations:** 1 Department of Physics, C-209 Howell Science Complex, East Carolina University, Greenville, NC 27858 USA; 2 Global Medical Consulting, LLC, Brentwood, TN 37027 USA

**Keywords:** Cancer imaging, Radiotherapy

## Abstract

Increasing interests in using magnetic resonance imaging only in radiation therapy require methods for predicting the computed tomography numbers from MRI data. Here we propose a simple voxel method to generate the pseudo-CT (pCT) image using dual-contrast pelvic MRI data. The method is first trained with the CT data and dual-contrast MRI data (two sets of MRI with different sequences) of multiple patients, where the anatomical structures in the images after deformable image registration are segmented into several regions, and after MRI intensity normalizations a regression analysis is used to determine a two-variable polynomial function for each region to relate a voxel’s two MRI intensity values to its CT number. We first evaluate the accuracy via the Hounsfield unit (HU) difference between the pseudo-CT and reference-CT (rCT) images and obtain the average mean absolute error as 40.3 ± 2.9 HU from leave-one-out-cross-validation (LOOCV) across all six patients, which is better than most previous results and comparable to another study using the more complicated atlas-based method. We also perform a dosimetric evaluation of the treatment plans based on pCT and rCT images and find the average passing rate within 2% dose difference to be 95.4% in point-to-point dose comparisons. Therefore, our method shows encouraging results in predicting the CT numbers. This polynomial method needs less computer storage than the interpolation method and can be readily extended to the case of more than two MRI sequences.

## Introduction

The workflow of conventional radiation therapy (RT) uses computed tomography (CT) images to create the treatment plan and to position the patient at treatment. Magnetic resonance imaging (MRI) is also often used to provide precise delineation of RT target volumes due to its superior soft tissue contrast^1^. When these two modalities are both used, the workflow then necessitates an additional step of registering the images of the two modalities: MRI and CT^2^. Recently, the concept of MRI-Linac using magnetic resonance imaging only in radiation therapy, i.e., MRI-only RT, has become more popular, because MRI-only RT has the benefits of improving the workflow and removing systematic errors in registering MRI and CT images^3,4^.

However, in MRI-only RT there is a problem in creating treatment plans with the MRI images because of the lack of CT images or the electron density information. To address this problem, various methods of generating CT images, called pseudo-CT (pCT), have been investigated^5–7^. Existing methods in deriving a pseudo-CT from MR images may be classified into different categories. They include the classification into segmentation-based, intensity-based, atlas-based, and hybrid methods^7^, or the classification into segmentation-, atlas-, patch-, and learning-based methods^8^, or the classification into bulk density assignment, atlas-based, and voxel-based methods^9^. These methods have produced mean absolute error (MAE) values ranging from 85 HU^10^ to 137 HU^11^ for the brain and from 36.5 HU^12^ to 74.3 HU^13^ for the prostate (pelvis region), for example.

Among these methods, atlas-based methods^12,14–17^ align an MRI atlas, which has been derived from an MRI database pre-registered to the corresponding reference-CT (rCT) images, to the target patient’s MRI images through registration. The atlas thus contains pre-determined correlations between the MRI voxels and the variables of interest such as the CT number or organ type. The same registration (with the translational, rotational, and deformable information) that maps the MRI atlas to the target patient’s MRI images is then applied to the atlas CT images to create the target patient’ pCT images. This approach is popular because of its potential in producing reliable pCT images with conventional MRI images. However, it requires accurate deformable image registration between the atlas and the target patient’s magnetic resonance (MR) images, which can be difficult, especially when large anatomical variations or pathological differences exist. This problem can be partially overcome by using multiple atlases or the hybrid method that combines the atlas method with other methods to increase the overall strength and reduce the overall weakness^18–20^.

Learning-based methods employ model-fitting or statistical learning techniques to generate a mapping function that correlates the MRI information to the corresponding CT numbers^9,21–28^. Deep learning is a machine learning technique that is useful for processing low-level noisy data such as medical images. Currently, a main deep learning technique used in the field of radiation oncology is the deep convolutional neural network that is based on the U-net architecture^24,26,28^. An advantage of learning-based approaches is that they can take into account neighborhood voxels^23,26^.

Voxel-based methods^9,11,13,23,29–35^, such as the bulk-density method^36,37^, mainly translate the voxel intensity information in the MRI images to CT numbers and pCT images. In general, a voxel-based method is specific to the MRI sequence(s) that the model is trained on but does not need the target patient anatomy to closely match the training patients. In contrast, atlas-based methods for generating the pCT do not depend on the MRI sequence(s) but depend on the patient anatomy, where a major difference between the target patient anatomy and the patient database population may result in inaccurate pCT images.

In this study, we investigate a voxel method that uses two MRI image sets acquired using different MRI sequences together with two-variable polynomial fitting functions to create pseudo-CT images. This method is validated by leave-one-out-cross-validation (LOOCV)^8,10,11,16,18,22^, applied to a sample of six patients. We evaluate our method via MAE between the pCT and rCT images and also by comparing the dose distributions of simulated RT plans created on the pCT and rCT images.

## Methods

### Patient groups

Patients with carcinoma of the cervix were prospectively studied with serial MRI and CT scans during RT on an IRB-approved imaging protocol granted by the University & Medical Center Institutional Review Board (UMCIRB) of the East Carolina University (ECU). Informed consents were obtained from patients with all identifier information removed. Collection of data and methods were carried out in accordance with the guidelines and regulations of UMCIRB. We selected 6 female patient cervical image datasets. The 6 datasets selected for this study have none of the following: significant visible artifacts, considerable co-registration errors, or large rotational transformation for aligning the MRI and CT images. This study has no effect on the treatment of these patients.

Specifically, the six patients in this study were staged clinically with the International Federation of Gynecology and Obstetrics criteria^38^, including physical examinations, chest radiograph, tumor biopsy, complete blood count, serum chemistries, intravenous pyelogram, and abdominal-pelvic computed tomography. There were 2 patients in Stage IB, 2 in Stage IIA, and 2 in Stage IVB (inguinal metastasis). Median age was 55 years (range 25–89). Two MRI scans were acquired on a Siemens Magneton Espree 1.5 T scanner. The T1-weighted MR sequence (MR_1_) was a fast low angle shot with different repetition time TR, an echo time TE = 4.53 ms, a flip angle FA = 70°, a matrix size of 320 × 320, and a voxel size of 0.81 × 0.81 × 5.0 mm^3^. The T2-weighted MR sequence (MR_2_) was a turbo spin echo with different repetition time TR, an echo time TE = 87 ms, a similar flip angle FA, a matrix size of 320 × 320, and a voxel size of 0.81 × 0.81 × 5.0 mm^3^. Both T1-weighted (T1w) and T2-weighted (T2w) MRI sets have more than 30 images per scan. In addition, a whole body CT scan using 120 kVp, various mAs values, a matrix size of 512 × 512 × 326, and a voxel size of 0.97 × 0.97 × 4.0 mm^3^ was acquired for each patient on a Siemens Biograph CT within one month of the acquisition of MRI. The detailed acquisition parameters for the MRI and CT scans of each patient are given in Supplementary Table S1.

### Pre-processing

We register the CT image set with the deformable setting in multi-modality registration on Velocity (Varian Medical Systems) using the MR_1_ image set as the reference image; the same is done to register the MR_2_ image set. The CT dataset is resampled to the same resolution as the MRI dataset: axial image dimension of 320 × 320 with the voxel size at $\approx$ 0.81 × 0.81 × 5.0 mm^3^. Because the MRI images of a given sequence have different image contrasts for different patients, MRI images were normalized^8,9,27,34,35,39,40^. Specifically, for the MRI datasets acquired with the same MRI sequence, e.g. MR_1_, we first find the average intensity value for all patients and that for a given patient, then their ratio is used as the patient-specific correction factor to multiply the voxel MR_1_ intensity values of this given patient, so that the average MR_1_ intensity values for all patients become the same. In addition, a binary mask is created by using a voxel intensity threshold combined with the edge detection function in MATLAB to eliminate air voxels and cover only the imaged subject to reduce the computational burden and improve the pCT accuracy^11,40–42^. Air voxels are set to a value of −1000 HU.

### Segmentation

**Figure 1:**
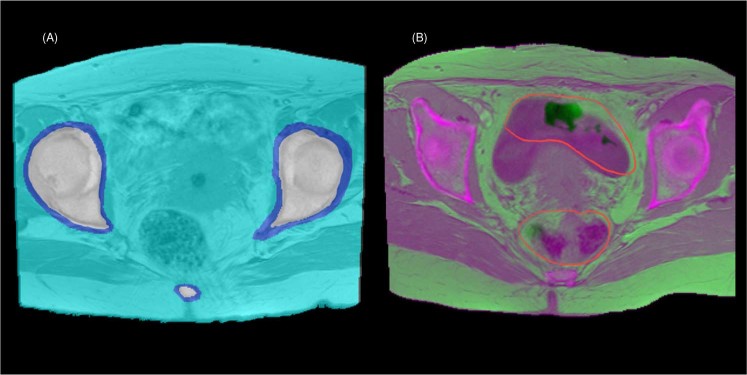
(**A**) Segmentation of the three regions superimposed on an MRI image: bony region (white), soft region (light blue), and mixed region (dark blue). (**B**) Example of a CT image (magenta) overlay on a corresponding MRI image (green) with the excluded region shown inside the red contours.

The imaged anatomical structure of each patient is manually segmented with masks into three regions: bony, soft, and mixed regions. An example of the three regions superimposed on an MRI image is shown Fig. 1A. The bony region contains the cortical and cancellous bone tissues, while the soft region contains all soft tissues. For the bony region, a mask is defined at the edge of the cortical bone with approximately 1 mm margin left out. A margin of approximately 2–3 mm surrounding the bony tissue is not included within the soft region. Margins are generated automatically by expansion or reduction of the mask. The mixed region represents the region between the above two margins. Then voxels within the same region are grouped together for determining the prediction model for the region.

When necessary, an excluded region, as shown in Fig. 1B, is drawn to select misaligned anatomical structures due in large part to changes during the elapse time between MRI and CT acquisitions. To obtain the correct correlation between MRI intensity values and the CT number of each voxel, voxels from the excluded region are not included in the training data used to determine or evaluate our prediction model.

### Modeling relation between two MRI intensity values and CT Number

The voxel method is based on the assumption that a relationship exists between a voxel’s CT number and MRI intensity value on average, at least for voxels of the same tissue type. However, voxels with the same MRI intensity value, even of the same tissue type, may have very different CT numbers. Therefore, our strategy is to have a second set of MRI images acquired with different MRI sequences, so that voxels with the same MRI intensity value (in the first set) but very different CT numbers may have different MRI intensity values in the second set, which will allow us to distinguish those voxels and map them to their corresponding CT numbers.

For each region of the segmented anatomical structures (bony, soft and mixed regions), all the voxels in the same region are grouped together as the training data to determine the prediction model for the region. The data from each voxel consists of its CT-value, the normalized intensity value ${S}_{1}$ from the MR_1_ image set, and the normalized intensity value ${S}_{2}$ from the MR_2_ image set. The prediction model is trained to map a voxel’s two MRI intensities to its CT number on the Hounsfield unit (HU) scale for each region. In this study, we used a two-variable *n*th-degree polynomial function that depends on a voxel’s two MRI intensity values to predict the voxel’s expected CT number:

1 $$\begin{array}{rcl}pCT({S}_{1},{S}_{2}) & = & \mathop{\sum }\limits_{{i}_{1},{i}_{2}\mathrm{=0}}^{{i}_{1}+{i}_{2}\le n}{c}_{{i}_{1},{i}_{2}}{S}_{1}^{{i}_{1}}{S}_{2}^{{i}_{2}}\\  & = & {c}_{\mathrm{0,0}}+{c}_{\mathrm{1,0}}{S}_{1}+{c}_{\mathrm{0,1}}{S}_{2}+{c}_{\mathrm{1,1}}{S}_{1}{S}_{2}+\ldots +{c}_{n\mathrm{,0}}{S}_{1}^{n}+{c}_{\mathrm{0,}n}{S}_{2}^{n}\mathrm{}.\end{array}$$

In practice, the MRI data of each region are separated into different MR_1_ and MR_2_ intensity value bins to reduce the computational burden and noise. An equal number of bins (Nbin) for MR_1_ and MR_2_ is used, while the bin width is determined based on the maximum normalized MR_1_ and MR_2_ intensity values (1,420 and 37,100 respectively in this study). As a result, every voxel is associated with two bin indices, e.g., *i* for the MR_1_ bin and $j$ for the MR_2_ bin, or $(i,j)$. Inside a given $(i,j)$ bin in these two dimensions, there can be multiple voxels with a range of CT numbers. We can define the average CT number of the bin as

2 $${\overline{CT}}_{i,j}=\frac{1}{{N}_{i,j}}\mathop{\sum }\limits_{k=1}^{{N}_{i,j}}C{T}_{i,j,k},$$

where ${N}_{i,j}$ is the number of voxels within the $i$-th MR_1_ bin and $j$-th MR_2_ bin, and $C{T}_{i,j,k}$ denotes the CT number of the $k-$ th voxel within this bin. We use equation (1) to perform a regression analysis on the training data of each region to determine the coefficients ${c}_{{i}_{1},{i}_{2}}$ in the polynomial, then $pCT({S}_{1},{S}_{2})$ best describes the average CT number ${\overline{CT}}_{i,j}$ as a function of the MRI intensity values ${S}_{1}$ and ${S}_{2}$. The regression analysis is performed by using the ‘NonlinearModelFit’ function in Mathematica (Wolfram, Champaign, United States). Note that a weighting factor based on the number of data points within a bin is used in the regression analysis. The 3-dimensional plots and contour plots of the polynomial function $pCT({S}_{1},{S}_{2})$ for each region of Cycle1 are provided in Supplementary Figures S1–S3 and S4–S6, respectively. We have also put the Mathematica source code of the pCT regression analysis along with example input files and the output file for the calculated polynomial coefficients at http://myweb.ecu.edu/linz/pCT/.

### Generation of pCT

To generate pseudo-CT images for a target patient, we first segment the anatomical structures on the MR_1_ and MR_2_ images of the target patient into the same types of regions: bony, soft, and mixed regions. For each region, the corresponding function from equation (1) is then applied to each voxel to map its ${S}_{1}$ and ${S}_{2}$ MRI intensity values to an expected CT number. Note that we restrict the predicted CT number to -1000 HU and 2000 HU. The pseudo-CT images are then obtained for the target patient after the CT number is generated for all the voxels. The total time to convert a whole pelvic MRI scan is within approximately 3 minutes using a Mac Pro (mid-2012) desktop with 2 × 2.4 GHz 6-Core Intel Xeon processor and 16 GB (8 × 2 GB) 1333 MHz DDR3 ECC memory.

To evaluate the accuracy of our method, we apply LOOCV for the six patients. There are six cycles, where five patients are used as the training data to determine the coefficients ${c}_{{i}_{1},{i}_{2}}$ in equation (1) for each region and then the prediction model is applied to the remaining (target) patient.

### Mean absolute error

To evaluate the quality of the generated pCT images from our model, we use the mean absolute error as defined below:

3 $$MAE=\frac{1}{N}\mathop{\sum }\limits_{k\mathrm{=1}}^{N}|pC{T}_{k}-rC{T}_{k}|,$$

where $N$ is the total number of body voxels (except voxels from the excluded region) of the target patient, $pC{T}_{k}$ and $rC{T}_{k}$ denote respectively the CT number from the generated pCT and the rCT for voxel $k$. The MAE thus measures the voxel-wise average error. When evaluated for each region, $N$ in equation (3) then represents the total number of body voxels in that region of the target patient.

### Optimization of parameters in the prediction model

**Figure 2:**
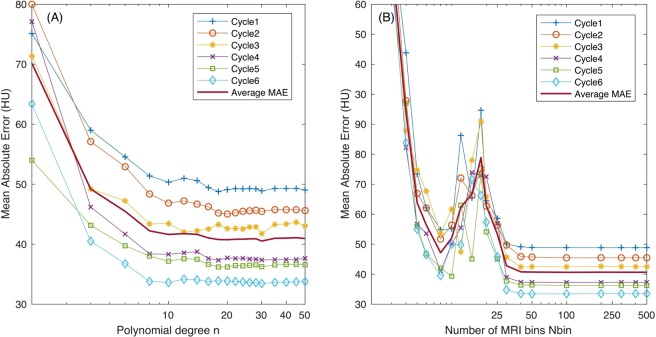
(**A**) Mean absolute error versus the polynomial degree $n$ with Nbin = 200. (**B**) Mean absolute error versus the number of MRI bins (Nbin) with the polynomial degree $n=30$.

We determine the optimal value of two key parameters in our prediction model: the polynomial degree $n$, and the number of MRI bins Nbin. From Fig. 2A, we see an overall decreasing trend in the average MAE value as the polynomial degree $n$ increases (with Nbin = 200). In particular, the model’s prediction improves as degree $n$ increases to $n\sim 20$, and further increase of $n$ changes the average MAE by no more than $\sim \mathrm{1 \% }$. Therefore, we use $n=30$ as the optimal value for the polynomial degree in equation (1). The effect of the number of MRI bins on the MAE values is shown in Fig. 2B. Although the MAE has a non-monotonous dependence on Nbin from ~10 to ~25, the average MAE values are the lowest for Nbin $\mathrm{ > 40}$ and shows little further decrease with Nbin. Since the computing burden of using Nbin of 200 versus Nbin of 40 is similarly small, and a polynomial function with a higher Nbin should better correlate the MR intensities to the CT number when the number of patients gets bigger in future studies, we choose to use Nbin = 200.

In addition, not all $(i,j)$ bins are filled, i.e., have voxels from the training data and thus have corresponding CT numbers. This often causes the polynomial function to rapidly deteriorate in the MRI region with empty bins. To improve the stability of the regression analysis, we fill the empty $(i,j)$ bins with CT numbers, thus all MRI $(i,j)$ bins have corresponding CT numbers before the regression analysis. In particular, we assign an empty bin with the CT number of the filled bin with the smallest distance. For example, for an empty bin with the MR_1_ bin number ${i}_{e}$ and MR_2_ bin number ${j}_{e}$, its distance from a filled bin with the MR_1_ bin number $i$ and MR_2_ bin number $j$ is calculated as

4 $$d=\sqrt{{(i-{i}_{e})}^{2}+{(j-{j}_{e})}^{2}}\mathrm{}.$$

The HU-value of the filled bin with the smallest distance $d$ is then assigned to the empty bin. If the smallest distance corresponds to multiple filled bins, then the average HU-value of those multiple bins is assigned to the empty bin. For our regression analysis, the weighting factor assigned to an empty bin is 10% of that of the nearest filled bin.

## Results

### Accuracy of the pCT image

**Figure 3:**
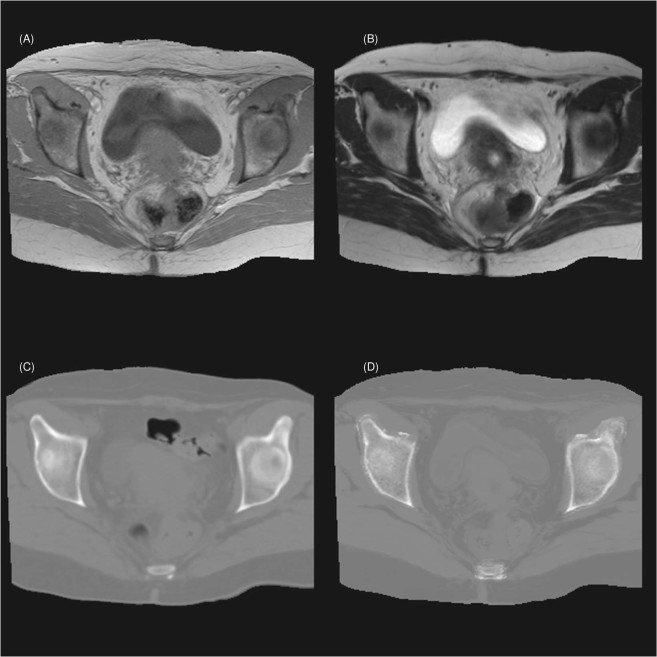
Axial views of the (**A**) MR_1_, (**B**) MR_2_, (**C**) reference-CT, and (**D**) generated pCT image at a given location of a target patient.

An example of the pCT image is shown in Fig. 3 with the axial view of the patient’s MRI (MR_1_ and MR_2_) and CT images at the same slice. We see that the pCT image (Fig. 3D) closely matches the rCT (Fig. 3C). Noticeable structural differences are predominantly in the soft tissues, specifically the bladder and bowel. Similar differences are also observed between the rCT (Fig. 3C) and MRI images (Fig. 3A,B). Other minor differences are observed at the muscle-fat tissue and bony-tissue interfaces. These can be mostly attributed to the extended duration between the acquisition times of MRI and CT images. Therefore, the region with major structural differences (e.g. bladder and bowel due to daily changes) between the CT and MRI images are segmented as the excluded region and omitted in determining the prediction model or the MAE calculations.

Table 1 presents the MAE values for the overall patient volume and for each segmented (bony, soft, mixed) region of each LOOCV cycle as well as the average value for all six cycles. We obtained an average MAE of 40.3 ± 2.9 HU for the overall volume in the images, while the raw MAE values for the bony, soft, and mixed regions are 102.7 ± 9.9 HU, 24.6 ± 1.0 HU, and 143.5 ± 9.2 HU, respectively. The weighted MAE value for a region is determined by multiplying the raw MAE value by the percent of voxels in that region, thus the sum of the weighted MAE values of the three regions equals the overall MAE value. We find that the bone-tissue interface has the most noticeable differences, because there the deviations between MRI and reference-CT images are the highest due to registration errors. The high raw MAE values for the mixed region in Table 1 support this observation, since the mixed region consists mainly of the bone-tissue interface. Also, because the bony region and the mixed region account for only a small percentage (9% and 7% respectively) of all the voxels in the imaged volume, their contributions to the overall MAE (i.e., their weighted MAE values) are lower than that from the soft region.

**Table 1:** MAE values between pseudo-CT and reference-CT for the overall patient volume and for each segmented (bony, soft, and mixed) region of each LOOCV cycle and their average values for all six cycles.

	Mean Absolute Error (HU)						
	Overall	Bony Region		Soft Region		Mixed Region	
		Raw	(Weighted)	Raw	(Weighted)	Raw	(Weighted)
Cycle1	44.4	118.5	(12.1)	25.8	(21.1)	138.3	(11.2)
Cycle2	42.7	100.2	(8.1)	25.6	(20.9)	134.3	(13.8)
Cycle3	40.9	104.0	(9.6)	24.8	(20.8)	151.8	(10.4)
Cycle4	38.7	105.6	(9.5)	23.0	(19.5)	157.0	(9.6)
Cycle5	38.1	99.7	(8.8)	24.6	(20.9)	135.8	(8.5)
Cycle6	36.7	88.0	(8.0)	23.9	(20.4)	143.6	(8.3)
Average	40.3 ± 2.9	102.7 ± 9.9	(9.4 ± 1.5)	24.6 ± 1.0	(20.6 ± 0.6)	143.5 ± 9.2	(10.3 ± 2.0)

The weighted MAE value of a region is the raw MAE value multiplied by the percent of voxels in that region among all voxels.

### Comparison of dosimetric calculations using pCT and rCT

To further assess the accuracy of the pCT image volume, we compare dosimetric calculations of treatment plans based on the pCT and the rCT. External beam pelvic radiotherapy with cisplatin and brachytherapy is the standard of care for patients with advanced cervical malignancy. The typical geometrical setup for cervical cancer is 4-field box technique, namely anterior-posterior (AP), posterior-Anterior (PA), right-lateral (RLAT) and left-lateral (LLAT) fields. A modified setup is a 3-field technique including RLAT, LLAT and AP, which can be used to reduce dose to rectum. In this study we use the 3-field treatment plan created with the Eclipse treatment planning system (Varian Medical System, Palo Alto, CA, USA), where the planning target volume (PTV) is the cervix. We then draw contours for the left and right femur heads, bladder, bowel, and rectum on the treatment planning system. For the excluded regions (mainly the bladder and bowel) where the CT and MR images differ significantly, the CT numbers for the bladder and bowel are overridden and assigned as 5 HU and 45 HU, respectively (for both the pCT and rCT). The center of the PTV is prescribed a dose of 56 Gy and for simplicity all three beams are equally weighted. The treatment plan is first generated on the rCT images and then mapped directly to the pCT images. Dose distributions of rCT and pCT treatment plans are calculated on the treatment planning system.

We compare the dose value of each voxel from the pCT and rCT dose distributions. A voxel that has a dose difference within the tolerance of $\mathrm{|2 \% |}$ is considered as passing the criterion. Passing voxels within the imaged volume are tabulated (not including the air voxels), and a passing rate is calculated. Table 2 shows the passing rate results, where the average passing rate is 95.4% in the point-to-point dose comparisons between the pCT and rCT treatment plans. When the pCT image is close to rCT, the dose distributions in a small region of interest would be very close to each other, which difference can be measured through the gamma index. Note that our point-to-point dose comparison method is similar to the gamma dose distribution comparison method except for the distance to agreement (DTA) value. In fact, our comparison method corresponds to using DTA = 0 and is thus stricter than the usual gamma index.

**Table 2:** Passing rates (i.e., with a dose difference less than 2%) from the point-to-point dose comparisons between the pseudo-CT and reference-CT treatment plans.

	Cycle1	Cycle2	Cycle3	Cycle4	Cycle5	Cycle6	Average
Passing rate	95.7%	94.8%	95.8%	96.2%	95.7%	94.2%	(95.4 ± 0.7)%

### Sources of the pCT uncertainties

Here we investigate in more detail the sources that contribute to the finite MAE between the generated pCT and the reference-CT images. The rCT image, pCT image, and the image of their absolute difference (|rCT-pCT | ) at four different slices for patient 1 are provided in Supplementary Figures S7–S10. In general, the MAE has two main contributions: one from the imperfect fitting of the average CT number ${\overline{CT}}_{i,j}$ as a function of the MRI bin numbers $(i,j)$ with equation (1), the other from the intrinsic fluctuation or spread of the CT numbers at given MRI bin numbers $(i,j)$ around the mean value ${\overline{CT}}_{i,j}$.

Instead of MAE, it is simpler for this purpose to examine the root-mean-squared (rms) difference between pseudo-CT and reference-CT:

5 $${\sigma }_{pCT}=\sqrt{\frac{1}{N}\mathop{\sum }\limits_{k\mathrm{=1}}^{N}{(pC{T}_{k}-rC{T}_{k})}^{2}}\mathrm{\ ,}$$

where $N$ is the total number of voxels in the imaged volume (not including the voxels in the excluded region), and $k$ is voxel index. When evaluated for each region, $N$ in the above equation then represents the total number of body voxels in that region in the imaged volume. The ${\sigma }_{pCT}$ values are presented in Table 3. Note that as expected ${\sigma }_{pCT}$ is always higher than (or equal to) the corresponding raw MAE value, which can be verified by comparing Tables 1 and 3. Also, the largest values are observed for bony and mixed regions, consistent with the fact that their raw MAE values shown in Table 1 are higher than that of the soft region.

**Table 3:** ${\sigma }_{pCT}$ values between pseudo-CT and reference-CT for the overall patient volume and for each segmented region of each LOOCV cycle and their average values for all six cycles.

	$${\sigma }_{pCT}$$ (HU)			
	Overall	Bony Region	Soft Region	Mixed Region
Cycle1	86.1	160.0	48.4	188.1
Cycle2	80.7	133.7	46.0	180.6
Cycle3	79.4	142.5	41.1	209.0
Cycle4	80.4	147.8	43.6	216.5
Cycle5	69.0	128.1	39.3	179.3
Cycle6	70.0	118.2	41.7	192.7
Average	77.6 ± 6.7	138.4 ± 14.9	43.3 ± 3.4	194.4 ± 15.3

To evaluate the goodness of fit of the polynomial equation (1), we can calculate the mean absolute error and the rms difference between the predicted $pCT({S}_{1},{S}_{2})$ from equation (1) and the averaged CT number ${\overline{CT}}_{i,j}$ in the reference-CT training data for each segmented region:

6 $$MA{E}_{fit}=\frac{1}{N}\sum _{i,j\mathrm{=1}}|{\overline{CT}}_{i,j}-pC{T}_{i,j}|{N}_{i,j}\mathrm{,\ \ \ \ \ }{\sigma }_{fit}=\sqrt{\frac{1}{N}\sum _{i,j\mathrm{=1}}{({\overline{CT}}_{i,j}-pC{T}_{i,j})}^{2}{N}_{i,j}},$$

where $N={\sum }_{i,j\mathrm{=1}}^{Nbin}{N}_{i,j}$ gives the total number of voxels in the imaged volume for that region. In the above equation, $pC{T}_{i,j}\equiv pCT({S}_{1},{S}_{2})$ is the predicted CT number for the MRI bin $(i,j)$, where ${S}_{1}$ and ${S}_{2}$ here represents the central intensity value of the $i$-th MR_1_ bin and the $j$-th MR_2_ bin, respectively. Table 4 shows the MAE_*fit*_ and ${\sigma }_{fit}$ values for each cycle and their average values. We can see that the fit is better for the soft region than for the other two regions.

**Table 4:** MAE_*fit*_ and ${\sigma }_{fit}$ values for fitting the mean reference-CT numbers with equation (1) for the overall patient volume and for each segmented region of each LOOCV cycle and their average values for all six cycles.

	MAE_*fit*_ (HU)			${\sigma }_{fit}$ (HU)		
	Bony Region	Soft Region	Mixed Region	Bony Region	Soft Region	Mixed Region
Cycle1	10.4	2.0	16.6	21.5	5.3	31.6
Cycle2	11.0	2.1	17.3	22.6	4.5	33.3
Cycle3	10.4	2.1	16.0	20.0	5.4	31.3
Cycle4	10.5	2.1	15.8	21.4	5.5	29.8
Cycle5	11.3	2.1	16.5	23.2	5.7	32.4
Cycle6	10.9	2.1	15.9	23.3	5.4	31.7
Average	10.8 ± 0.4	2.1 ± 0.0	16.3 ± 0.6	22.0 ± 1.3	5.3 ± 0.4	31.7 ± 1.2

Another contribution to the MAE of the generated pCT comes from the fact that voxels at the same MRI bin numbers $(i,j)$ do not have exactly the same CT number, although they average to the mean value ${\overline{CT}}_{i,j}$. For this, we can calculate for each region the mean absolute error and the rms difference between individual voxel’s reference-CT number and the averaged value ${\overline{CT}}_{i,j}$ at the same MRI bin $(i,j)$ as

7 $${{\rm{MAE}}}_{rCT}=\frac{1}{N}\mathop{\sum }\limits_{i,j\mathrm{=1}}^{Nbin}\mathop{\sum }\limits_{k\mathrm{=1}}^{{N}_{i,j}}|rC{T}_{k}-{\overline{CT}}_{i,j}\mathrm{|,\ \ \ \ \ }{\sigma }_{rCT}=\sqrt{\frac{1}{N}\sum _{i,j\mathrm{=1}}\mathop{\sum }\limits_{k\mathrm{=1}}^{{N}_{i,j}}{(rC{T}_{k}-{\overline{CT}}_{i,j})}^{2}}\,,$$

where N = ${\sum }_{i,j\mathrm{=1}}^{Nbin}{N}_{i,j}$ and $k$ is voxel index within the MRI bin $(i,j)$. These values are shown in Table 5. We see that the values for the bony region and the mixed region are much higher than that of the soft region, similar to Table 3.

**Table 5:** MAE_*rCT*_ and ${\sigma }_{rCT}$ values for the spread of CT numbers in the same MRI bin for the overall patient volume and for each segmented region of each LOOCV cycle and their average values for all six cycles.

	MAE_*rCT*_ (HU)			${\sigma }_{rCT}$ (HU)		
	Bony Region	Soft Region	Mixed Region	Bony Region	Soft Region	Mixed Region
Cycle1	95.3	23.4	138.0	128.4	40.2	187.5
Cycle2	97.6	23.7	141.3	132.5	40.3	190.6
Cycle3	97.6	23.7	133.3	131.9	41.4	183.6
Cycle4	96.8	24.1	133.3	129.9	41.0	182.7
Cycle5	98.8	23.6	138.1	134.4	42.5	189.3
Cycle6	100.3	23.9	135.1	135.3	41.2	186.8
Average	97.7 ± 1.7	23.7 ± 0.2	136.5 ± 3.2	132.1 ± 2.6	41.1 ± 0.8	186.7 ± 3.1

We also find that the ${\rm{M}}A{E}_{rCT}$ or ${\sigma }_{rCT}$ values are much higher than the corresponding ${\rm{M}}A{E}_{fit}$ or ${\sigma }_{fit}$ values for the same region. Furthermore, if the uncertainties from these two sources were independent, one would expect the following relation for each region:

8 $${\sigma }_{pCT}^{2}={\sigma }_{rCT}^{2}+{\sigma }_{fit}^{2}\mathrm{}.$$

From Tables 3–5, we indeed see that the average ${\sigma }_{pCT}$ value of each region is higher than the corresponding average value of ${\sigma }_{rCT}$ or ${\sigma }_{fit}$, and the above relation is approximately satisfied even quantitatively. Similar features can also be observed for the MAE values from Tables 1, 4 and 5. Specifically, the average raw MAE value of each region is higher than the corresponding average value of ${\rm{M}}A{E}_{rCT}$ or ${\rm{M}}A{E}_{fit}$, with ${\rm{M}}A{E}_{rCT}\gg {\rm{M}}A{E}_{fit}$, and the relation ${\rm{M}}A{E}^{2}\approx {\rm{M}}A{E}_{rCT}^{2}+{\rm{M}}A{E}_{fit}^{2}$ holds approximately. Therefore, we conclude that the intrinsic fluctuation of the reference-CT numbers for voxels with similar MRI intensity values, not the fitting procedure from the regression analysis, is the dominant source to the uncertainty of the generated pCT images.

## Discussions

We have presented a voxel method that uses two sets of MRI images acquired with different MRI sequences to generate the pCT images. This method is straightforward to implement and extend. For example, this method can be easily generalized to use more than two sets of MRI contrast data, where equation (1) would just include additional independent MRI variables. Our method differs from earlier pCT works^11,13,23,29–35^, mainly in the use of high-degree polynomials with more than one MRI variables together with the use of deformable image registration and MRI intensity normalizations.

Using multiple MRI sets with different sequences allows for improved delineation and identification of voxels with different CT numbers and thus leads to better accuracy of the pCT. Table 6 compares our MAE values with those using only one set of MRI image (MR_1_ or MR_2_), including the corresponding two-sided p-values from the paired t-test to compare the ‘Current Method’ to the single-MRI methods. The small p-values (p < 0.001 and p = 0.0026) suggest that our method of using dual-contrast MRI data improves the pCT accuracy.

**Table 6:** MAE values from our method using two MRI sets with segmentation, in comparison with those using only one MRI set, using the interpolation method, using two MRI sets but without segmentation, without using the excluded region, and using only 4-patient datasets.

	Mean Absolute Error (HU)						
	Current Method	Only MR_1_	Only MR_2_	Interpolation	Without Segmentation	Without Using Excluded Region	LOOCV
							3 + 1
Cycle1	44.4	48.2	55.6	44.5	66.0	45.3	48.5
Cycle2	42.7	47.3	64.4	42.9	65.3	47.4	
Cycle3	40.9	46.1	81.5	41.1	61.9	43.1	40.7
Cycle4	38.7	44.1	64.6	38.8	60.8	47.4	39.7
Cycle5	38.1	41.7	53.2	38.3	64.7	40.8	
Cycle6	36.7	40.8	66.4	36.9	59.5	41.0	37.2
Average	40.3 ± 2.9	44.7 ± 3.0	64.3 ± 10.0	40.4 ± 2.9	63.0 ± 2.7	44.1 ± 2.9	41.5 ± 4.9
p-value		<0.001	0.0026	<0.001	<0.001	0.017	0.25

The p-value is the two-sided value determined by performing a paired t-test between a given method and the ‘Current Method’ for sample size 6 (except that the sample size is 4 for the t-test of ‘LOOCV 3 + 1’).

We note that the use of multiple MR sequence parameters has been explored earlier and the advantage of using more than one MRI sequence has been pointed out^8,30^. Aouadi *et al*.^16^ used a patch-based method for the brain and also combined T1- and T2-weighted MRI intensities to have an enhanced description of tissue properties. Burgos *et al*.^17^ used an atlas-based method to generate pCT images, where each subject had a T1w MR image, T2w MR image, and a CT image for pelvis acquired on the same day. Note that a disadvantage of the atlas method is that it will be unable to extrapolate to a feasible pCT image without pre-existing templates for other anatomic areas or an atypical anatomy. Speier *et al*.^34^ investigated the generation of pCT images from T1w and T2w MRI images for the brain including a voxel-based method that used a localized lookup table, where the lookup table algorithm relied on segmentation and regionalization steps in the data preprocessing. Pileggi *et al*.^35^ used T1w and T2w MR for brain to generate a pCT for proton therapy treatment, where a voxel-based lookup table was generated by binning HU in matrixes of 10 × 10 MR intensity units together with rigid image registration. Koike *et al*.^9^ described a method to generate pCT images from T1w, T2w and fluid-attenuated inversion recovery MR images using an adversarial network for the head region. Zhong *et al*.^8^ used a patch-based approach and reported a MAE of 97.72 ± 15.78 HU for combined T1w and T2w training but 113.73 ± 16.86 HU for only T1w, showing an improvement in the pCT accuracy from using both T1w and T2w MR sets.

In addition to using a polynomial to calculate the average CT number as a continuous function of the two MRI variables, we have also used an interpolation method that is similar to using a lookup table. We first use the center points of all the MR_1_-MR_2_ bins to divide the MR_1_-MR_2_ plane into ~Nbin^2^ number of cells. Then we calculate the average CT number of an arbitrary point in the MR_2_-MR_2_ plane by using a bilinear interpolation of the average CT numbers of the four neighboring center points. Table 6 shows that the MAE values from this interpolation method are only slightly higher than those from our current method. The p-value (p < 0.001) is small because the paired MAE differences between the interpolation method and our current method from Table 6 (0.1, 0.2, 0.2, 0.1, 0.2, and 0.2 HU, respectively) all have the same sign and are close in magnitude, even though the mean MAE difference is very small (~0.17 HU). We note that, compared to using a lookup table^34,35^, using a continuous polynomial function to represent the complicated relation between the MR values and the corresponding CT value has an advantage in that the computer storage of such a polynomial function needs less space since only the coefficients are needed. This advantage in storage will be even greater if more than two MRI sequences or contrasts will be used. We also note that other methods, such as probability functions^19,30^, the gaussian mixture regression model^11,22^, and weighted summation^13,33^, have also been used in other studies to calculate the CT number from the input MRI data.

Similar to other studies^10,29,31,43^, setting masks to segment tissues is an important step in improving the pCT accuracy. The importance of segmentation can be seen from the ‘Without Segmentation’ column of Table 6, where MAE values obtained without segmentation (still with the same excluded region and using two MRI sets) are significantly higher. The small p-value (p < 0.001) from the paired t-test suggests that the inclusion of segmentation in the approach of deriving pCT improves its accuracy. Furthermore, we believe the implementation of an automated segmentation process^33^ to our method will not only further improve the efficiency but also further improve the accuracy and robustness of our method by ensuring better agreements in the region delineation between patients.

One issue of this study was that the MRI and CT patient data could not be acquired on the same day. This created uncertainties during the image registration process due to the rectal/bladder filling inconsistency between sessions and other setup inconsistencies. To address this issue, we have used a deformable image registration and the excluded region. The excluded region as shown in Fig. 1B contains mainly of organs, bowel and bladder that could have significant internal motion and anatomical differences on a daily basis^44–46^. When we include voxels in the excluded region by applying the corresponding fitting functions, we get the MAE values in the ‘Without Using Excluded Region’ column of Table 6. The small p-value (p = 0.017) from the paired t-test suggests that using an excluded region improves the pCT accuracy. The implementation of better alignment and a shorter duration between the acquisition of CT and MRI datasets into our method could help in reducing the size of the excluded region and thus improve the pCT accuracy. Note that the excluded region is not needed when applying the prediction model to the actual MRI data to generate the pCT images of the target patient.

Since our datasets of 6 patients are rather limited, we investigate the effect of sample size by applying LOOCV for 4 out of the 6 datasets (i.e., assuming that we only have data for patient number 1, 3, 4, and 6). The ‘LOOCV 3 + 1’ column of Table 6 shows the corresponding MAE values of using three training datasets and one dataset as the target patient in generating the pCT, which are close to the MAE values of our current ‘LOOCV 5 + 1’ method. We also conduct the paired t-test to compare the MAE values from ‘LOOCV 3 + 1’ with the corresponding values from our ‘Current Method’ (i.e., the MAE values under ‘Current Method’ for Cycle1, 3, 4, and 6). The rather large p-value (p = 0.25) indicates that there is insufficient evidence to conclude there is a significant difference between the MAE of ‘LOOCV 5 + 1’ and ‘LOOCV 3 + 1’. However, to bring our proposed method to implementation in the clinical routine, we would like to have more training patients (than five used in this study) and also have the MRI and CT data of a given patient acquired on the same day.

In the pre-processing, a deformable image registration^12,19,47^ is used to ensure that the anatomical position of each voxel between the MRI and the CT images matches and anatomical structures between the image modalities are aligned. Matching of anatomical positions is necessary in training our model to establish the relationship between the MRI voxel intensity values and the CT number while suppressing the noise from mismatched voxels. For generating the pCT for the target patient, however, a deformable registration will not be necessary, thus the geometric integrity of the target patient images is retained.

We could further improve this model by determining the optimal MRI sequences for acquiring the dual-contrast MRI data. We also recommend using the same MRI sequence parameters to acquire each set of the dual-contrast MRI data (for the training patients as well as the target patient); then the MRI intensities of different patients would be more consistent, which would further improve the pCT accuracy. Note that different anatomical locations (e.g., lung or head and neck) from the pelvic region used in this study could possibly require different optimal MRI sequences for the MRI acquisition, therefore extending the method into different anatomical sites is warranted.

In conclusion, we have developed a voxel-based method that uses two different MR sequence sets (dual-contrast MRI) to create pseudo-CT images, where after deformable image registration and MRI intensity normalizations a regression analysis is used to determine the two-variable high-degree polynomial function for each segmented region. Using pelvic data from six patients, the HU values and dose distributions from the pseudo-CT are in close agreements with those from the reference-CT. Therefore, the pseudo-CT generation using a multi-variable polynomial prediction model with deformable image registration, anatomical segmentation and MRI intensity normalizations shows promising results for MRI-only radiation treatment planning. Our proposed polynomial method is easy to extend to more MRI sequences and saves computer storage. In addition, its accuracy can be further improved in the future by optimizing sequence parameters of the dual-contrast MRI or by using MRI data with more than two different sequences.

## Supplementary information

Supplementary information.

## Data Availability

All data generated and/or analyzed during the current study are available from the corresponding author on reasonable request.
