# Supplementary material for: Generation of Pseudo-CT using High-Degree Polynomial Regression on Dual-Contrast Pelvic MRI Data
Source: Sci Rep. 2020 May 15;10:8118. doi: 10.1038/s41598-020-64842-3 (PMC7229007; doi:10.1038/s41598-020-64842-3)
Supplement: Supplementary file 1 — Supplementary information. [file 41598_2020_64842_MOESM1_ESM.pdf]

Generation of Pseudo-CT using High-Degree Polynomial Regression on Dual-Contrast Pelvic MRI Data

Samuel C. Leu[1], **Zhibin Huang**[1,2], **and Ziwei Lin**[1,*]

[1]Department of Physics, C-209 Howell Science Complex, East Carolina University, Greenville, NC 27858, USA
[2]Global Medical Consulting, LLC, Brentwood, TN 37027, USA
[*]Correspondence and requests for materials should be addressed to Z.L. (linz@ecu.edu)

ABSTRACT

Supplementary materials are provided below.

	MR_1 (T1-weighted)			MR_2 (T2-weighted)			CT	
	TR (ms)	TE (ms)	Flip angle (FA)	TR (ms)	TE (ms)	Flip angle (FA)	kVp value	mAs value
Patient 1	150	4.53	70°	2870	87	180°	120	176
Patient 2	150	4.53	70°	3060	87	180°	120	228
Patient 3	150	4.53	70°	3720	87	180°	120	153
Patient 4	150	4.53	70°	3060	87	180°	120	119
Patient 5	130	4.53	70°	3820	87	180°	120	132
Patient 6	177	4.53	70°	3060	87	160°	120	166

Supplemental Table S1. Parameters for the MR and CT scans of each patient.

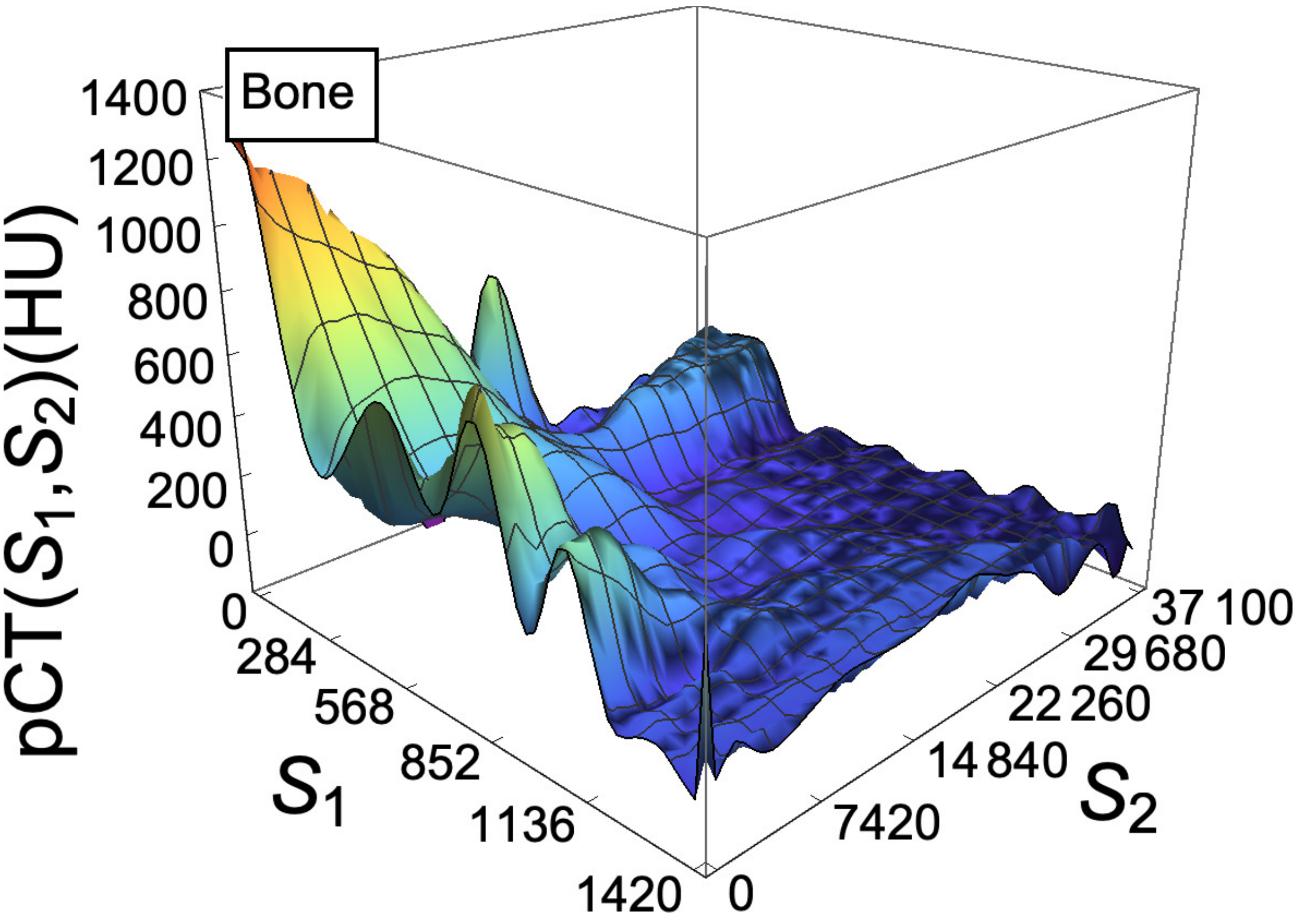

Supplemental Figure S1. Three-dimensional plot of the polynomial function $pCT(S_1, S_2)$ for the bony region of Cycle1.

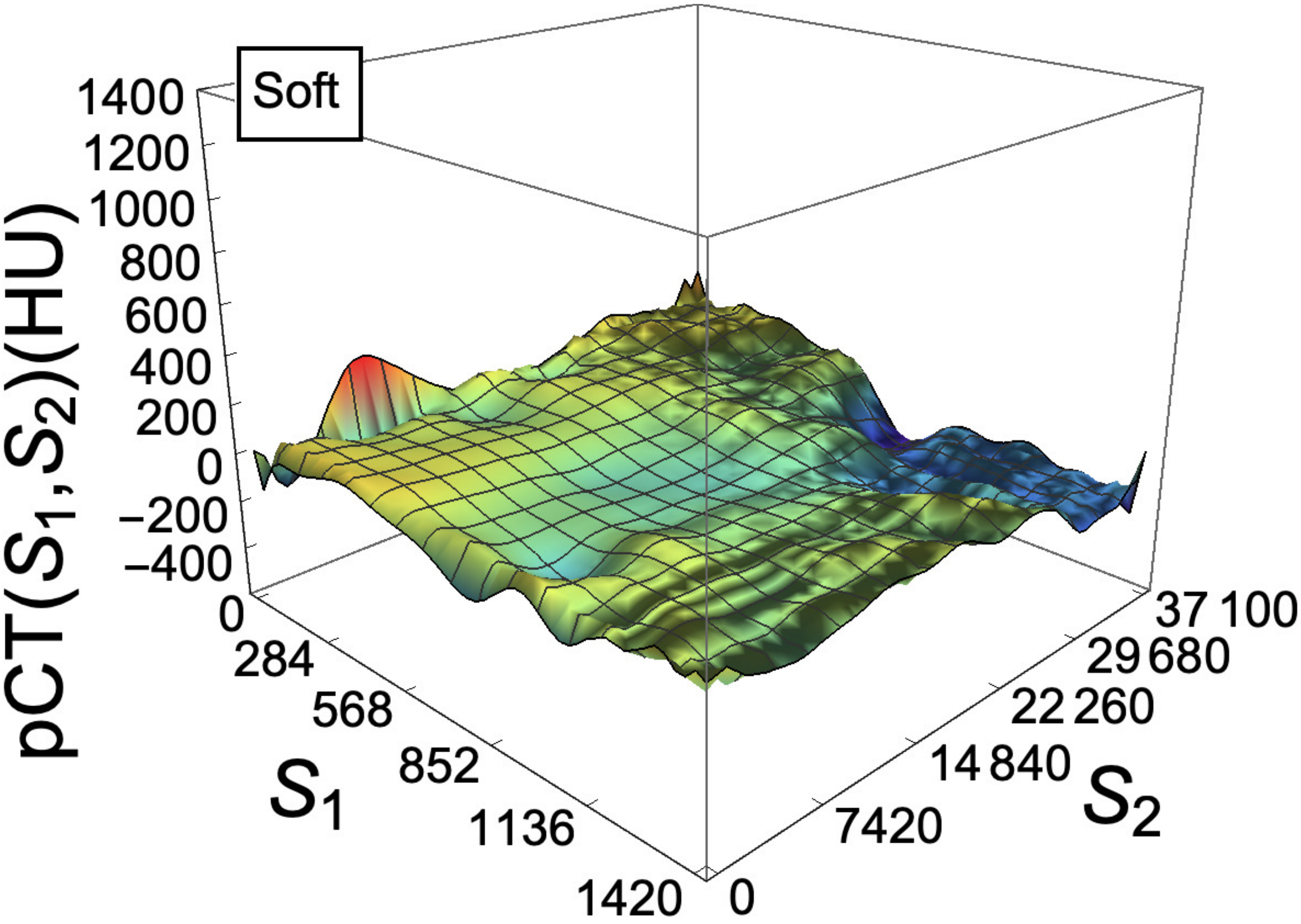

Supplemental Figure S2. Same as Fig. S1 but for the soft region.

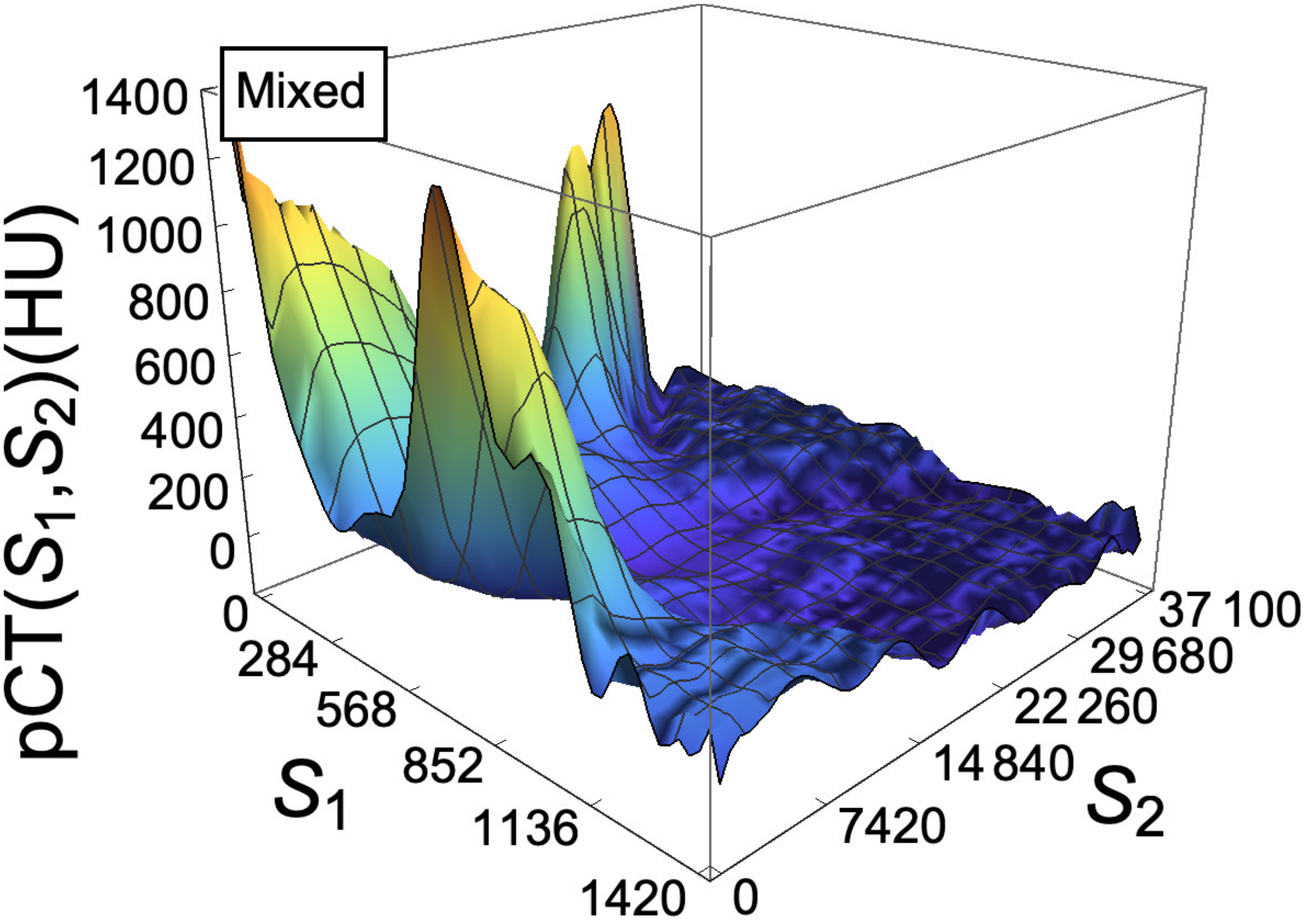

Supplemental Figure S3. Same as Fig. S1 but for the mixed region.

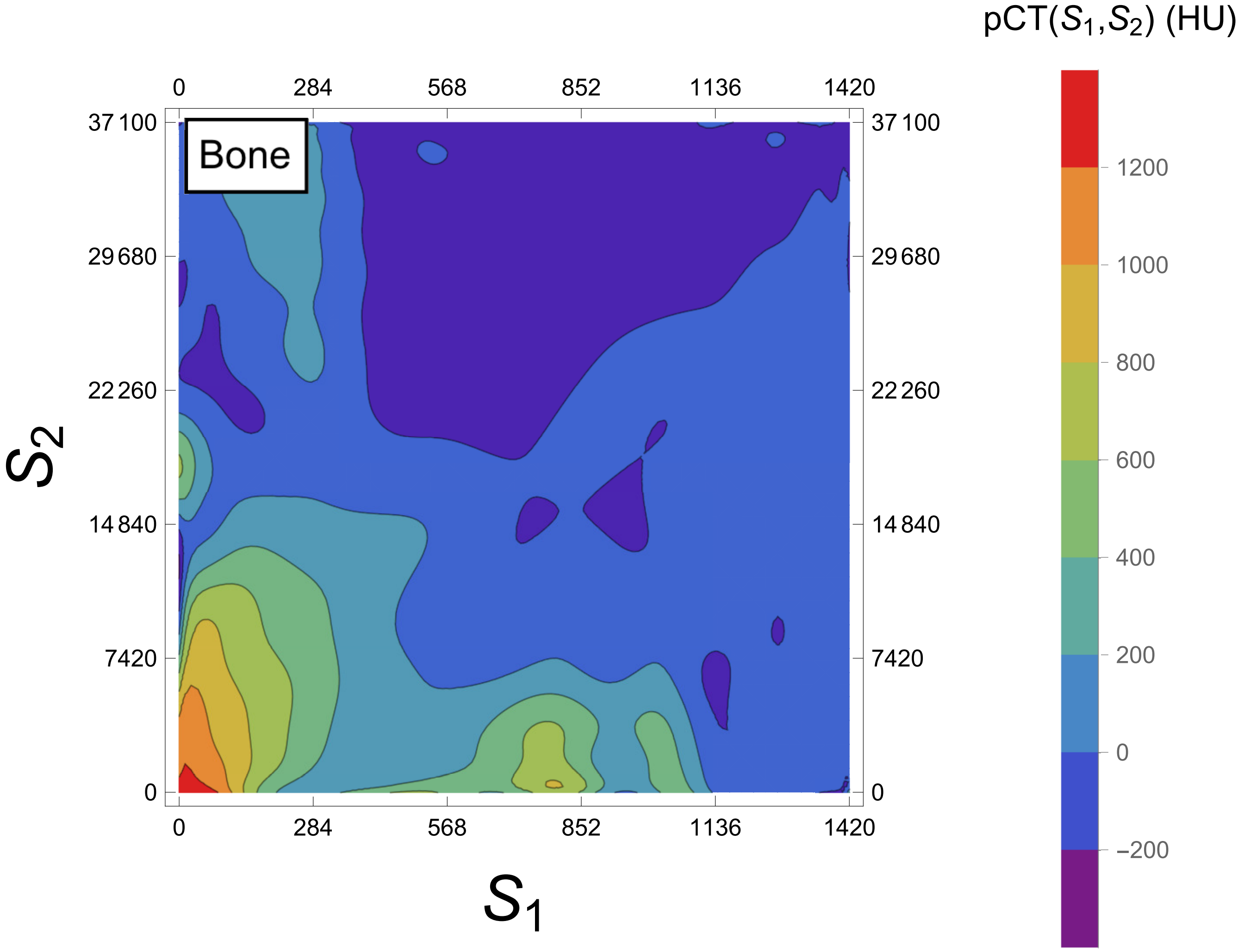

Supplemental Figure S4. Contour plot of the polynomial function $pCT(S_1, S_2)$ for the bony region of Cycle1.

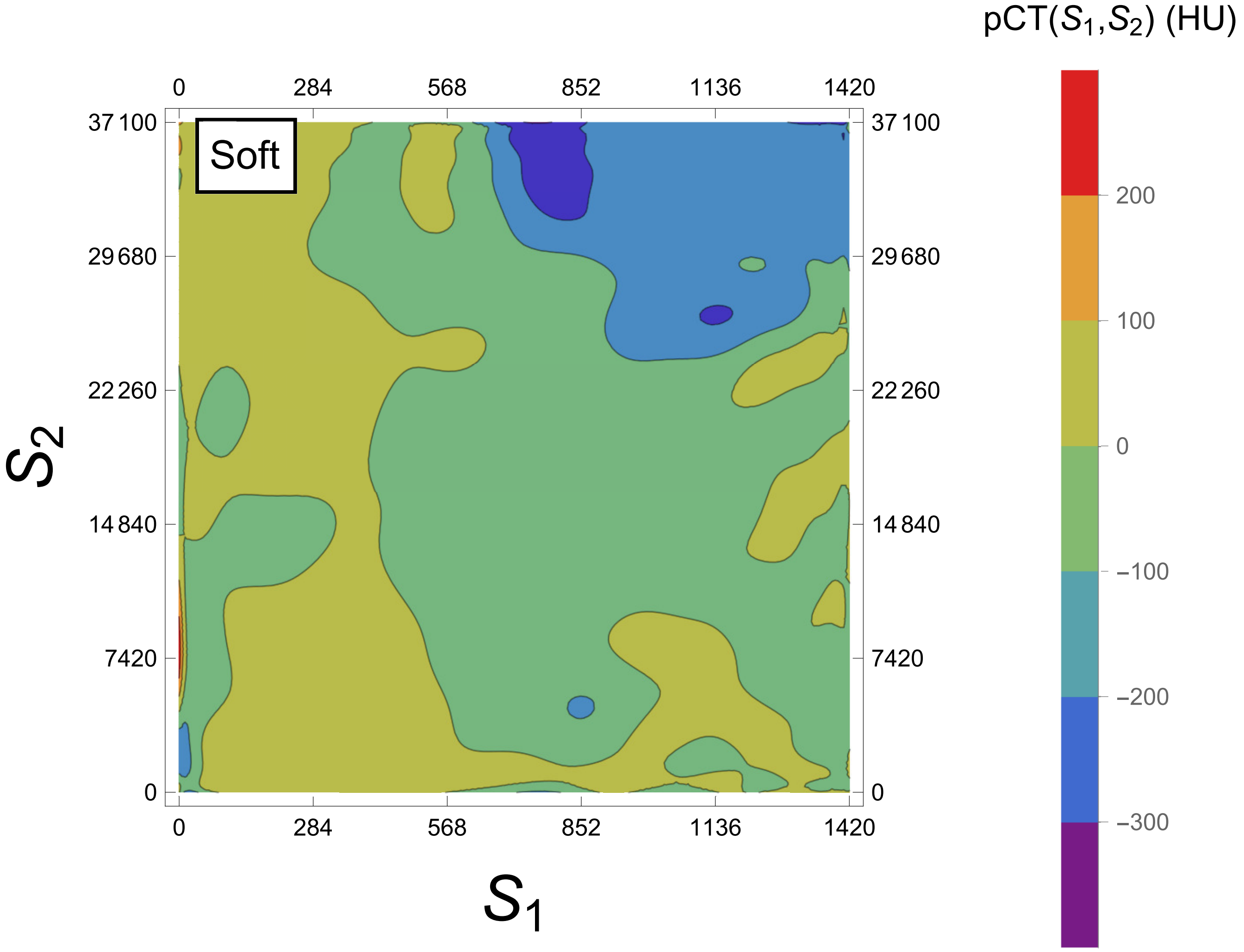

Supplemental Figure S5. Same as Fig. S4 but for the soft region.

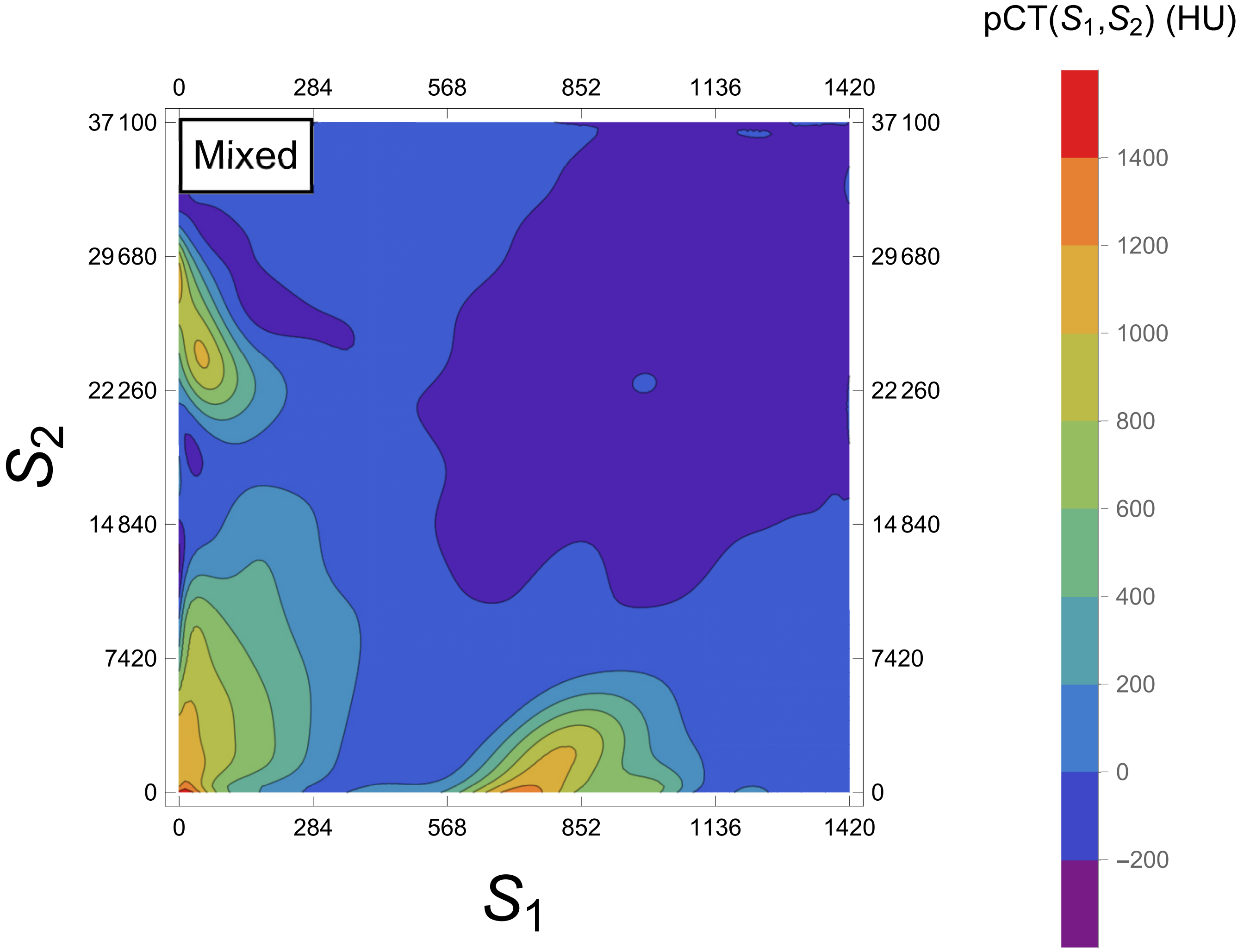

Supplemental Figure S6. Same as Fig. S4 but for the mixed region.

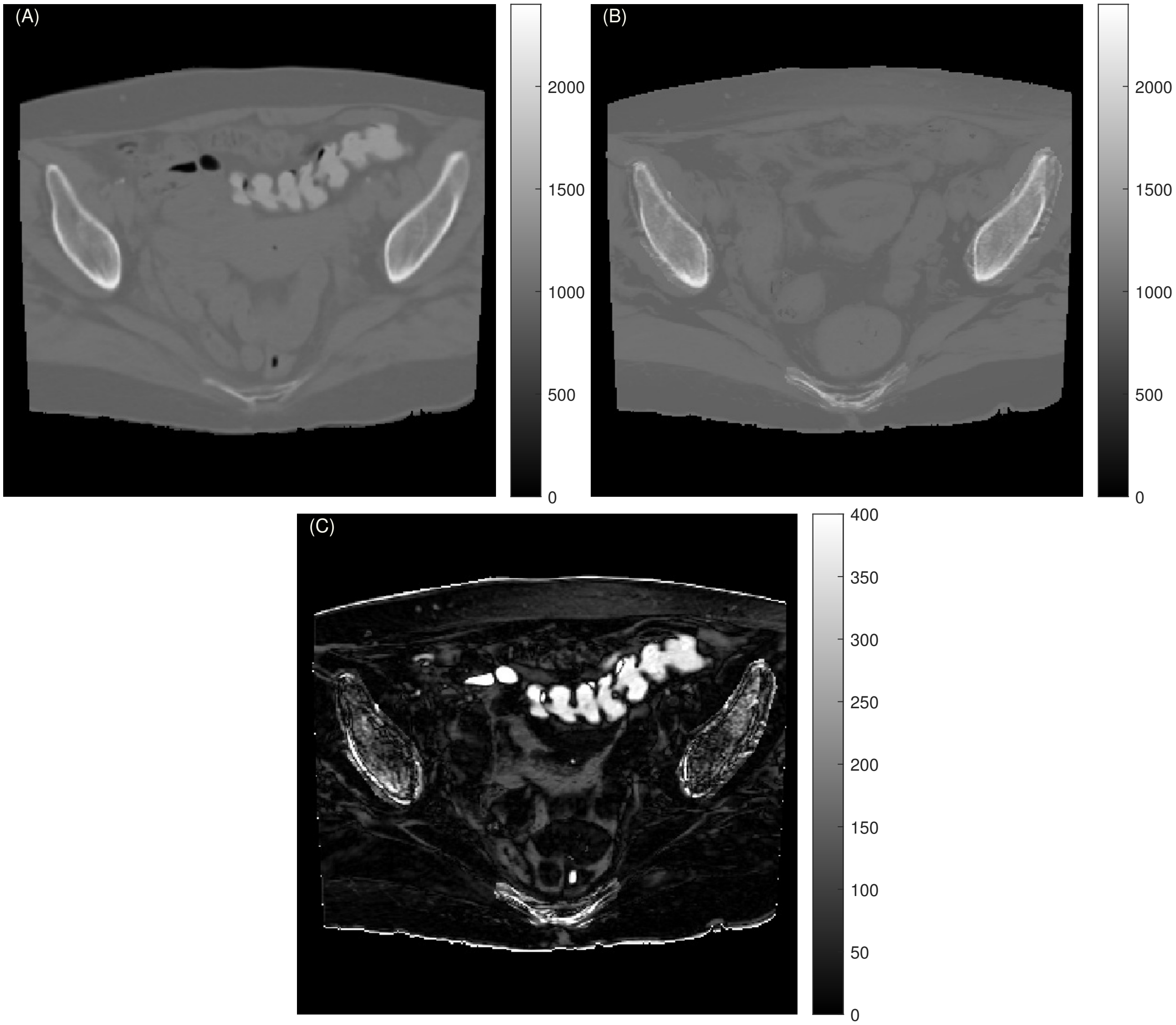

Supplemental Figure S7. (a) The rCT image, (b) pCT image, and (c) the image of their absolute difference (|rCT-pCT|) at slice #8 for patient 1. Note that, due to the long duration between MRI and CT acquisitions, there are changes in anatomy such as the upper high density area in the bowel region; this region is within the excluded region and thus not included in either the training data or the MAE calculation.

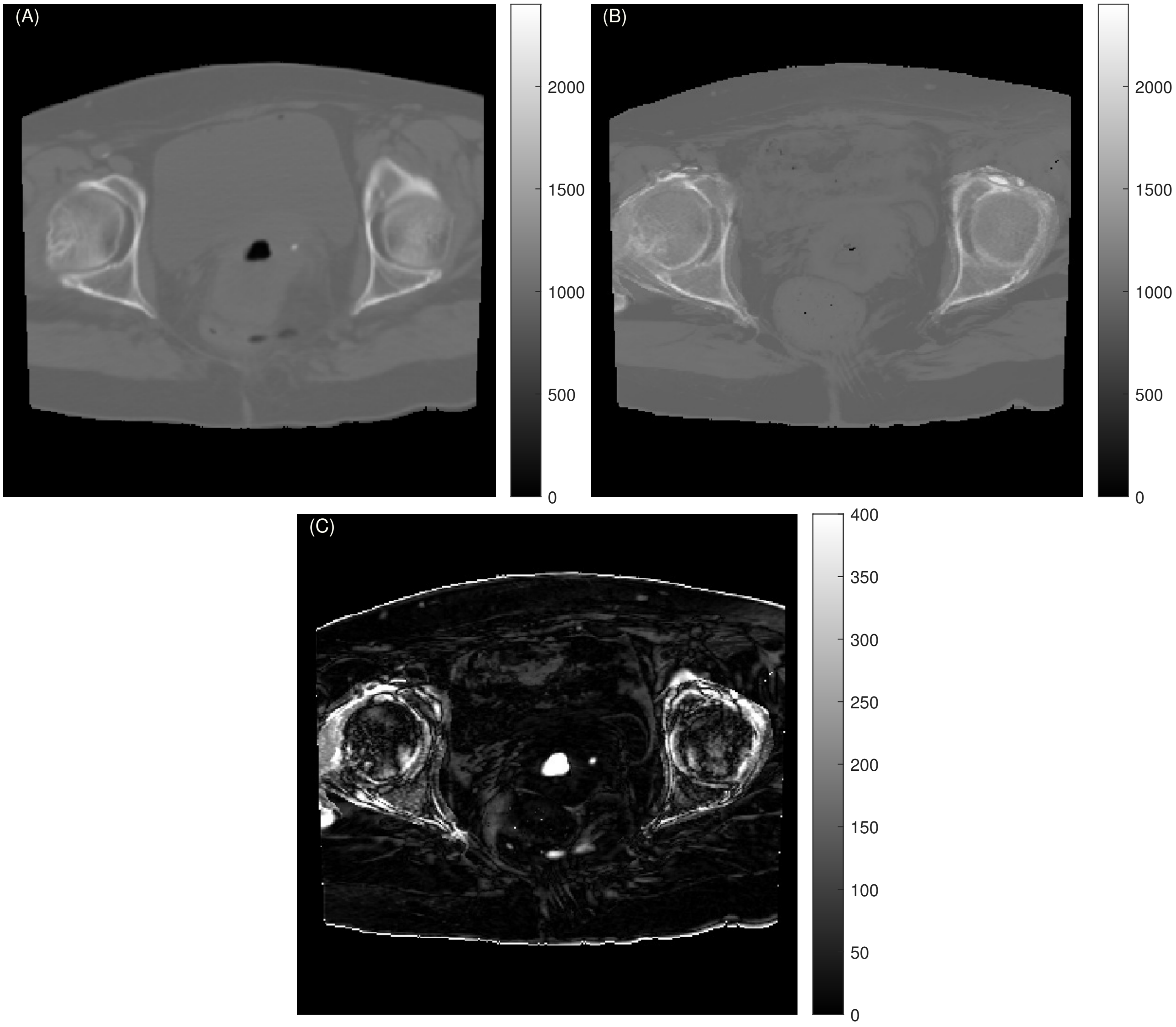

Supplemental Figure S8. (a) The rCT image, (b) pCT image, and (c) the image of their absolute difference (|rCT-pCT|) at slice #15 for patient 1.

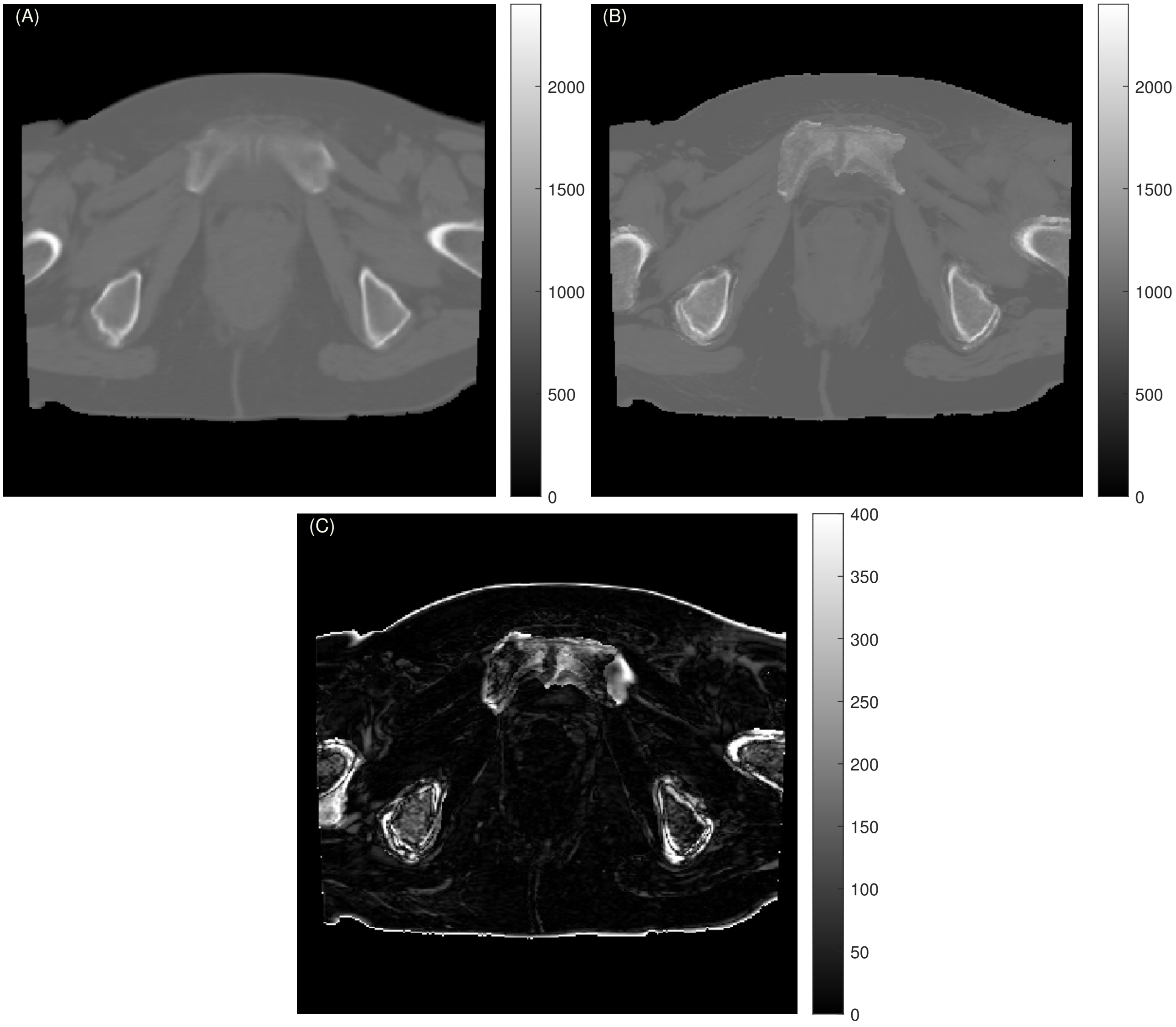

Supplemental Figure S9. Same as Fig. S8 but for slice #22.

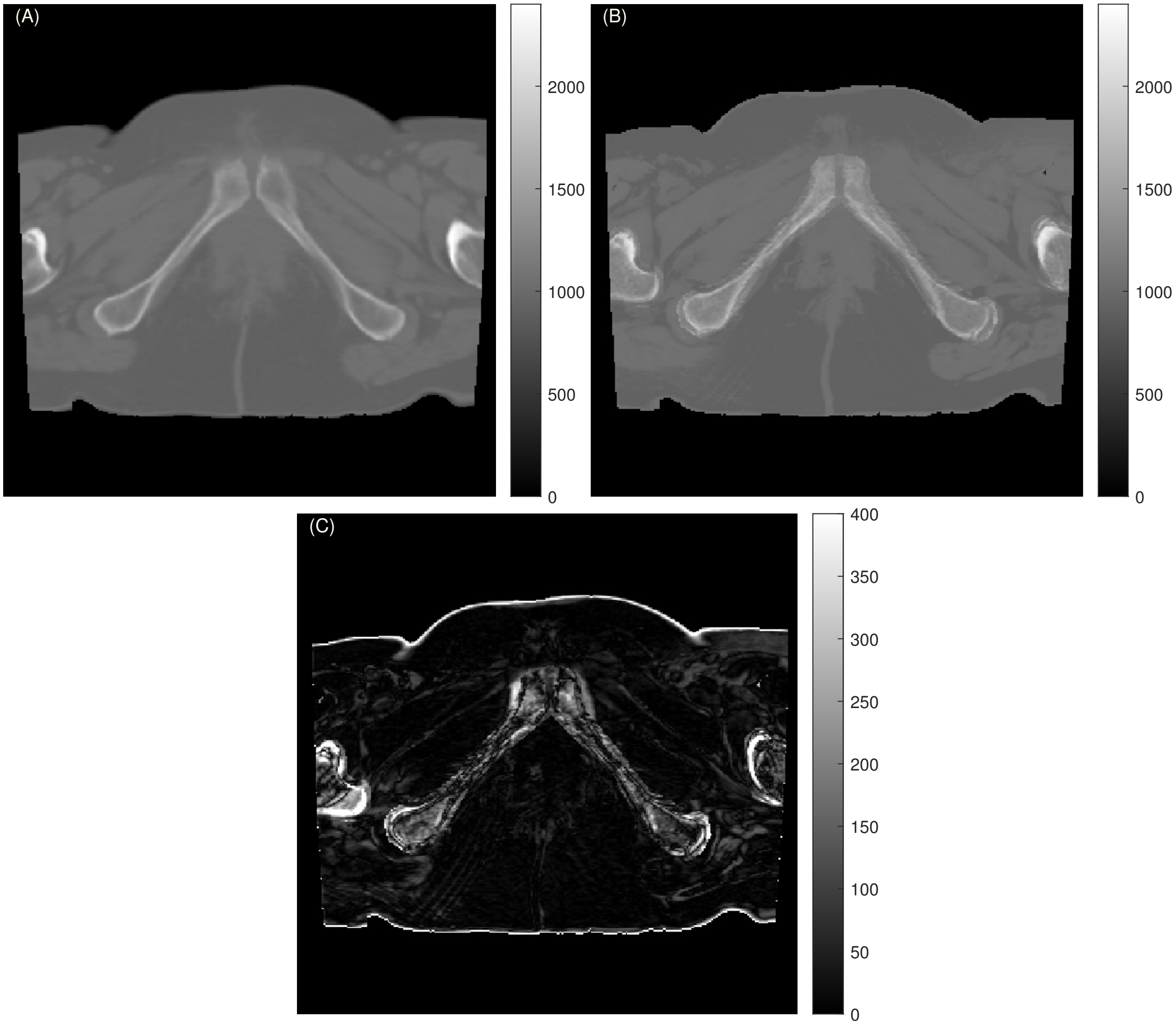

Supplemental Figure S10. Same as Fig. S8 but for slice #26.
